# Regeneration and transient gene expression in protoplasts of *Draparnaldia* (chlorophytes), an emerging model for comparative analyses with basal streptophytes

**DOI:** 10.1186/s13007-019-0460-6

**Published:** 2019-07-12

**Authors:** Lenka Caisová, Timothy O. Jobe

**Affiliations:** 1 Centre for Plant Sciences, Faculty of Biological Sciences, University of Leeds, Woodhouse Lane, Leeds, LS2 9JT UK; 2 Botanical Institute, Cluster of Excellence on Plant Sciences (CEPLAS), University of Cologne, Zülpicher Str. 47b, 50674 Cologne, Germany

**Keywords:** Chlorophytes, Colonization of land, *Draparnaldia*, Land plants, Model organism, Protoplasts, Streptophytes, Transformation

## Abstract

**Background:**

Green plants comprise two lineages: (1) the streptophytes that colonised land and (2) the chlorophytes that have adaptations to land but remained mostly aquatic. To better understand what made streptophytes so successful, we are currently establishing the chlorophyte alga *Draparnaldia* sp. (Chaetophorales, Chlorophyceae) as a model for comparative analyses between these two lineages. However, establishing *Draparnaldia* as a valuable model requires that it can be transformed. Thus, our goal is to develop a transformation protocol for this alga.

**Results:**

We have established the first transformation protocol for *Draparnaldia*. This protocol is based on protoplast transformation by electroporation. It includes instructions on protoplast isolation, regeneration and transient transfection. It also provides a list of the effective selective agents for future *Draparnaldia* transformations.

**Conclusions:**

Our protocol opens a way for *Draparnaldia* functional genomics analyses. Moreover, it also provides an important base for establishment of stable transformation.

**Electronic supplementary material:**

The online version of this article (10.1186/s13007-019-0460-6) contains supplementary material, which is available to authorized users.

## Background

Colonization of land by plants was a major transition on Earth. Although it is generally accepted that land plants evolved from freshwater streptophyte algae, their key properties enabling such a transition are still poorly understood [1–5 and citations therein]. To examine these properties several basal land plant and streptophyte algal models, such as *Anthoceros* [6], *Chara* [7]*, Closterium* [8]*, Klebsormidium* [9], *Marchantia* [10], *Mougeotia* [11] and *Physcomitrella* [12] are (or are currently being) established. However, there are also many chlorophytes (a sister lineage to streptophyte algae and land plants) that moved to terrestrial habitats and morphologically even resemble mosses. This raises the important question of why no land plants have evolved from chlorophytes?

**Fig. 1:**
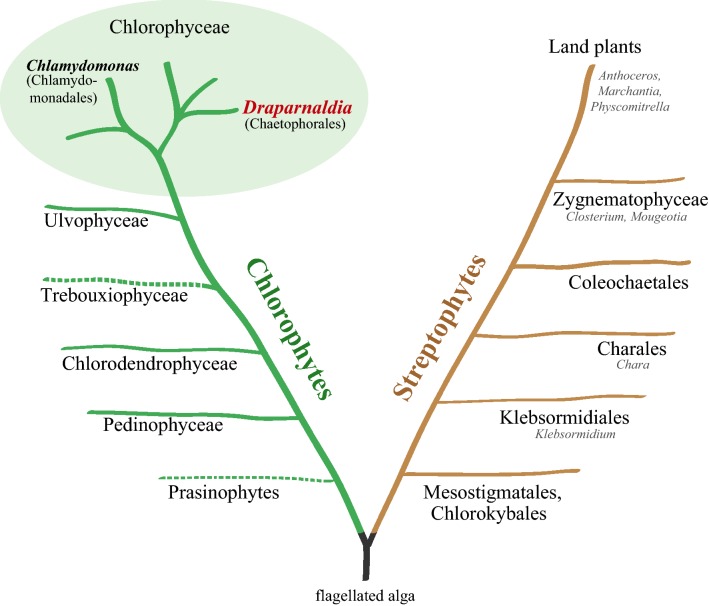
Phylogenetic position of *Draparnaldia* in the green tree of life. In addition to *Draparnaldia*, a well-established *Chlamydomonas* chlorophyte model species as well as several streptohyte models are shown. The tree schematic is based on Ref. [4, 65–67]. Dash-lined lineages are probably not monophyletic

**Fig. 2:**
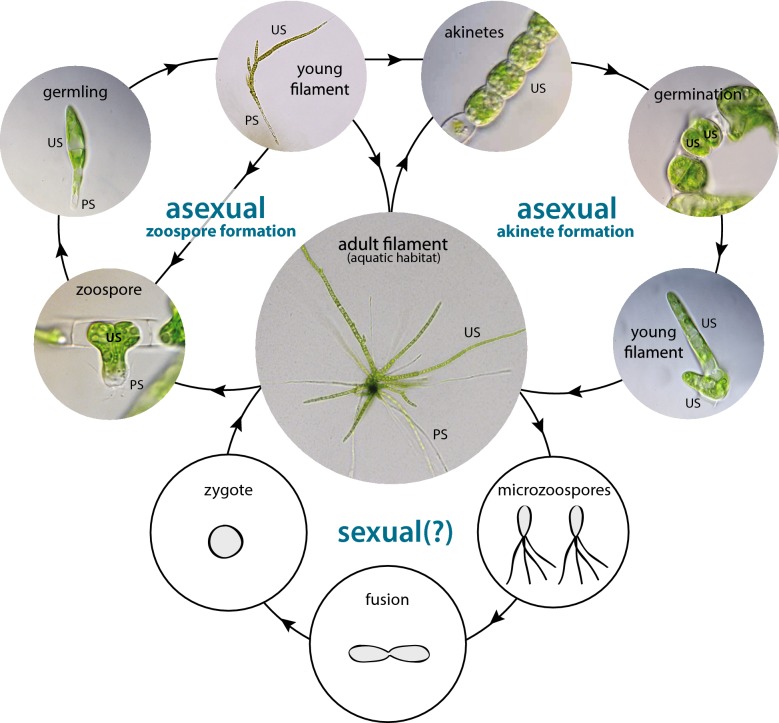
Life cycle of *Draparnaldia*. Two types of asexual reproduction are presented. Zoospores are strictly aquatic reproductive stages and have a distinct Upright Sytem (US) and Prostrate System (PS). Akinetes (= resting stages) are zoospores arrested in a parental filament. They are formed during drought periods and enable a long-term survival in the terrestrial habitat. However, water availability is required for their germination. The germinating akinetes have only the US, the PS is formed later. Note, that *Draparnaldia* is also capable of fragmentation (not shown). Fragmentation is initiated with algal transition from the aquatic to the terrestrial habitat. It leads to the filament splitting into two new filaments with a fully developed UP and PS. A general mechanism of the fragmentation process has been described in Ref. [29]. In addition, a sexual reproduction of *Draparnaldia* has been reported [68], but it has not been confirmed for this specific strain

To better understand what made streptophytes so successful, we are currently establishing the freshwater multicellular chlorophyte alga *Draparnaldia* sp. (Chaetophorales, Chlorophyceae) as a model for comparative analyses between these two lineages. For the phylogenetic position of *Draparnaldia* in the green tree of life see Fig. 1. *Draparnaldia* possesses a broad range of adaptations to aquatic and terrestrial habitats. It displays complex morphology similar to mosses and some streptophyte algae: branching filaments, rhizoids with apical growth, and tissue specialization [13–15]. It also reproduces in a similar manner as many streptophyte algae, for *Draparnaldia* life cycle see Fig. 2. Moreover, it is well positioned phylogenetically. It belongs to the Chaetophorales (fig. 1 in Ref. [13]), whose species range from unbranched filaments with a single-celled attachment to branched filaments with multi-celled rhizoids. Thus, it enables comparison of complex filamentous body development between chlorophytes and basal streptophytes. All these features make *Draparnaldia* an attractive model to distinguish properties that are unique to streptophytes from those that are common to both chlorophytes and streptophytes.

*Draparnaldia* transcriptome has recently been sequenced and will be published in a separate paper. In addition, there is a plan for genome sequencing. However, establishing *Draparnaldia* as a valuable model also requires that it is genetically transformable. From the variety of methods for plant and algal transformation [11, 16–21] we focused on transformation via protoplasts. The reasons for this choice were threefold: (1) It permits regeneration of the alga from a single cell, which is crucial for developmental studies. (2) Exogenous DNA can be delivered into the cell using different methods, such as electroporation [22, 23] or polyethylene glycol (PEG)-mediated transformation [24, 25]. (3) A few reports about protoplast isolation and regeneration in filamentous chlorophytes exist [26–28], suggesting that protoplast transformation might be possible. Here, we present the first protocol for protoplast transformation of *Draparnaldia*. The protocol consists of four parts: protoplast isolation, regeneration, transient transfection via electroporation, and identification of effective selective agents for future *Draparnaldia* transformations.

## Methods

### Chemicals and equipment

All chemicals were of highest purity grade and were purchased from Bayer, Duchefa Biochemie, Merck, New England BioLabs, Roth, Serva, Sigma-Aldrich or Thermo Fisher Scientific (Additional file 1: Supplement 1a). Equipment list with suppliers is provided in Additional file 1: Supplement 1b.

### Reagent setup

d-Mannitol, 0.5 M was prepared one day before use. 9.1 g of d-mannitol was dissolved in 100 mL of dH_2_O. The pH was adjusted to 7.2 with NaOH and/or HCl. The solution was sterilized with a 0.2-μm filter and stored at the room temperature.

Driselase, 2.5% stock solution was prepared just before use. 0.25 g driselase was dissolved in 10 mL of 0.5 M Mannitol solution (in 15 mL falcon). After that it was vortexed and wrapped with the aluminum foil, incubated on a shaker (40 rpm) for 30 min at 4 °C. This was followed by its centrifugation (2500 × *g* for 10 min) and filter sterilization using a 0.2-μm filter.

Regeneration medium 10 and 1 (RM10 and RM1) was derived from Growth medium (GM) by adding d-mannitol and calcium chloride (Table 1). Their names refer to the final concentration of calcium chloride. For RM10, 10 mL of Stock solution 1 was mixed with 196.6 μL of Stock solution 2. For RM1, 9 mL of Stock solution 1 was mixed with 1 mL of RM10. The media and stock solutions can be stored at 4 °C for at least 2 weeks.

**Table 1:** Stock solutions for RM10 and RM1

No.	Component	Stock solution	Stock concentration	Final concentration in RM10	Final concentration in RM1
1	d-Mannitol in GM	6 g/100 mL GM Dissolve, adjust pH to 6.6 with NaOH/HCl, autoclave.	0.33 M	0.33 M	0.33 M
2	CaCl_2_·2H_2_O	2.94 g/40 mL dH_2_O Dissolve and autoclave.	0.5 M	10 mM	1 mM

### *Draparnaldia* origin

The algal strain used in this study was *Draparnaldia* sp. CCAC 6921. The strain originates from a dry bank of the river ‘Rio Picocca’ in Sardinia, Italy. It was isolated from a leaf surface. The strain was identified by sequencing of the nuclear rRNA genes (18S, 5.8S, ITS2 and partial 28S; 2397 bp) according to Ref. [29]. The resulting sequence was most closely related to *Draparnaldia glomerata* CCAP 418/2, from which it differed by 2 nucleotides in ITS2 and 28S rDNA. The newly determined sequence is available under the accession number LR597279, Project: PRJEB33155 from the European Nucleotide Archive (ENA), [30]. The axenic strain of *Draparnaldia* sp. CCAC 6921 can be purchased from the Central Collection of Algal Cultures (CCAC; University of Duisburg-Essen).

### *Draparnaldia* cultivation

*Draparnaldia* sp. was cultivated axenically using aerated liquid culture. The growth medium (GM) was based on Bold’s basal medium [31]. Four vitamins were added, Vitamin B_12_ (0.6 μg/L), (+)-Biotin (3 μg/L), Thiamine·HCl (300 μg/L), Niacinamide (0.3 μg/L). The pH was adjusted to 6.6. Both filaments and protoplasts were grown under standard conditions as described previously [13]. A general guide for *Draparnaldia* cultivation, including the long-term storage and the recipe for GM, is provided in Additional file 2.

### Protoplast isolation

Protoplasts were isolated according to the protocol originally developed for *Physcomitrella patens* [16], with several modifications. The modified version of the protocol is described in the Results.

### Protoplast regeneration

The protocol for protoplast regeneration in liquid medium was developed in three steps.

1. *Regeneration Medium (RM).* GM was supplemented with mannitol for osmotic stabilisation of protoplasts and with calcium chloride to promote cell wall regeneration and subsequent division. The optimal concentration of mannitol was determined stepwise. Initially, *Draparnaldia* filaments were exposed to different concentrations of mannitol (0.2–0.5 M). This revealed that filaments can grow only below 0.35 M mannitol. Therefore, the lowest mannitol concentration capable of maintaining viable protoplasts was determined and chosen as the optimum. For CaCl_2_, the optimal concentration was determined by resuspending protoplasts in a 6-well culture plate containing 3 mL/well of RM comprising GM, mannitol and one of six concentrations of CaCl_2_ (0.17, 1, 2.5, 5, 7.5, 10 mM). This range was selected because 0.17 mM is the concentration used in GM of *Draparnaldia* and 10 mM is one of the highest concentrations used in protoplast regeneration of higher plants [32]. Protoplasts were observed daily. Selection criteria for the optimal calcium chloride concentration included > 85% survival rate of protoplasts, cell wall formation (visualized using the Calcofluor white staining [29], cell division, filament formation and branching.
2. *Optimal plating density for regeneration.* Protoplasts were grown at 4 different densities (10^2^, 10^3^, 10^4^, 10^5^ protoplasts/mL) using the RM developed in step (1). These values reflect optimal densities found across many algal and plant species [33–35]. Morphological observations were made 5, 10 and 20 days after protoplast inoculation. The plating density was considered ‘optimal’ when all surviving protoplasts were able to regenerate into branched filaments.
3. *Filament regeneration on GM.* RM was beneficial for early phases of regeneration. However, the osmoticum prevented restoration of the typical *Draparnaldia* morphology. To enable full regeneration, filaments were grown in RM until they began to branch (5–7 days after protoplast isolation). Then, the replacement of RM by GM was initiated. To this end, two strategies were tested. First, RM was completely replaced by GM. Second, RM was replaced gradually by removing 50% of the RM and adding the same volume of GM on the day when the first branching occurred. The same procedure was repeated again on the following day.

### Protoplast transfection

**Fig. 3:**
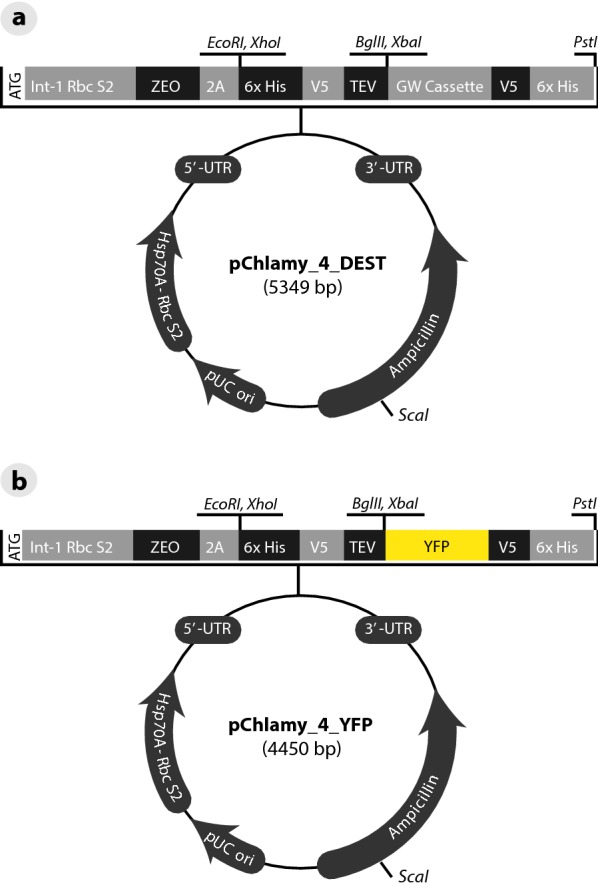
Modification of the *Chlamydomonas* expression plasmid pChlamy_4. a Purified pChlamy_4 was restriction digested with BamHI and KpnI followed by removal of overhangs with mungbean nuclease. A Gateway cassette was then ligated in frame with the self-cleaving FMDV 2a polyprotein linker to produce pChlamy_4_DEST. **b** eYFP was PCR amplified from pSAT6-eYFP-N1 using directional primers containing flanking attB sites and inserted into pDONR/Zeo. eYFP has not been codon optimized for *Chlamydomonas*. A LR recombination was performed with pDONR/Zeo-eYFP to create pChlamy_4_YFP

To transform *Draparnaldia* protoplasts, an electroporation protocol developed for the green alga *Chlamydomonas reinhardtii* was adapted [36]. For a detail protocol of how to electroporate *Draparnaldia* protoplasts see “Results”. In brief, to visualize protein expression, the expression plasmid pChlamy_4 [36] was modified to create the YFP-reporter construct pChlamy_4-eYFP (Fig. 3). This plasmid was selected because *Chlamydomonas* and *Draparnaldia* are closely related (see Fig. 1) and thus it was more likely that their regulatory elements will be conserved. Before electroporation the plasmid was linearized by ScaI digestion. The electroporation was performed in 0.4-cm electroporation cuvette with a 2 mm gap using electrical pulses of 300, 400 or 600 V. Each pulse was about 2 ms. Confocal microscopy of protoplasts was performed 3–10 days after electroporation. Images were collected on a Leica TCS-SPE confocal microscope (Leica Microsystems, Exton, PA USA) using a 63 × oil immersion objective. YFP was excited with the blue argon ion laser (488 nm), and emitted light was collected between 546 and 583 nm. Chloroplasts were excited with a 561 nm laser, and emitted light was collected from 570 to 651 nm. The two channels were collected separately, and later superimposed. Bright field images were collected simultaneously with the fluorescence images using the transmitted light detector. Images were processed using Leica Suite X software (version 3.3.0.16799).

### Determination of selective agents

Ten selective agents (including zeocin—a selective marker in pChlamy_4 plasmid) were tested in *Draparnaldia* (see “Results”). The initial screening was performed with 4 concentrations (5, 25, 50, 100 μg/mL), representing low and high values typically used for plants and algae [e.g. 25, 37–39]. For those agents that were able to kill *Draparnaldia*, the minimum inhibitory concentration was determined. Experiments used 15-day old regenerated filaments (original plating density 1200 protoplasts/mL) growing in liquid GM in 24-well culture plates. One of the wells was a positive control (GM without selective agents). The percentage of surviving (i.e. green) filaments was examined 7 and 14 days after the application of selective agents.

### Data collection and evaluation

All experiments were conducted in three replicates. All observations, except the transformation (see above), were performed directly in the 6- or 24-well culture plates. For establishment of the protoplast regeneration protocol, at least 200 protoplasts or filaments per well were examined. For survival tests of electroporated protoplasts and determination of effective selective agents, all filaments per dish/well were analysed. Standard deviations are shown in each graph and table.

## Results

### Protoplast isolation

**Fig. 4:**
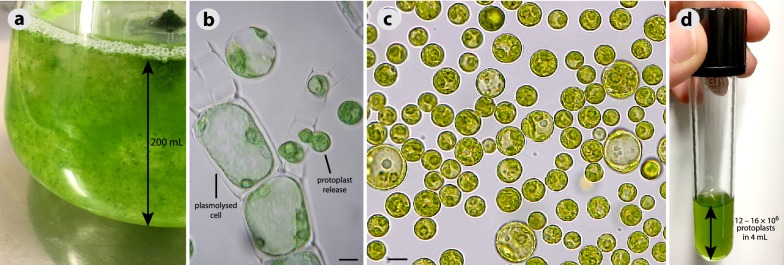
*Draparnaldia* protoplasts. a 7-day-old aerated culture, material sufficient for 2–3 isolations. **b** Cell plasmolysis and protoplast release. **c** Purified protoplasts. **d** Protoplast yield from a single isolation. Scale bar = 10 μm

First, we modified the Cove et al. (2009) protocol for protoplast isolation [16]. This included three major changes: (1) We did not grow the alga on agar, because it yielded only 10^3^ protoplasts/g of treated biomass. Instead, we used a liquid aerated culture (Fig. 4a), which yielded 10^6^ protoplasts/g of treated biomass. (2) We induced cell plasmolysis prior to cell wall digestion (Fig. 4b). This step improved viability of isolated protoplasts from approximately 70–95%. (3) We added two more purification steps, because the original protocol did not allow to separate protoplasts from cell debris and short filaments. The modified protocol is provided below. It takes 3–4 h and it yields between 12–16 × 10^6^ protoplasts per a single isolation (Fig. 4c, d).

#### Protocol for protoplast isolation

If not otherwise indicated, all steps should be done on a clean bench. All material used for protoplast isolation must be sterile to avoid contamination.

1. Before starting, prepare the biomass, material, media and reagents. For instructions of how to grow the liquid aerated culture of *Draparnaldia* see Additional file 2.
2. Harvest 3–4 g of fresh biomass of 7–10-day-old aerated culture using the polyester (PET) mesh (pluriSelect) with a pore size of 40 μm.
  Note: 3–4 g of biomass corresponds approximately to 60–90 mL of culture, depending on the density. 1 g of fresh biomass yields between 3–4 × 10^6^ protoplasts/mL.
3. Transfer the harvested biomass into a Petri dish (100 × 20 mm) using forceps.
4. To introduce plasmolysis, resuspend the biomass in 12 mL 0.5 M mannitol and seal the Petri dish with Parafilm.
5. Incubate the Petri dish for 35–40 min on rotator (70 rpm shaking, room temperature).
6. While waiting, prepare 2.5% driselase solution. For instructions see “Methods” section.
7. Add 4.5 mL of sterile driselase to the Petri dish with biomass and mannitol. Seal the Petri dish with Parafilm and cover it with aluminium foil to protect protoplasts from strong light.
  Note: The final concentration of driselase is > 0.68%.
8. Incubate the Petri dish with the mixture on a rotary shaker (30–40 rpm shaking, room temperature) until the majority of biomass breaks down. This takes about 45–60 min.
  Note: Protoplast isolation from older biomass (approximately 15 days old aerated culture) is possible, but not recommended. It takes 2–3 h longer and it is less efficient. It yields about 10^3^–10^4^ protoplast/g of biomass. The same holds true for non-aerated *Draparnaldia* cultures.
9. Check the viability of protoplasts under the light microscope. Abundant free-floating protoplasts and remnants of undigested biomass should be present.
  Note: An inverted microscope for tissue cultures (e.g. CK × 4, Olympus, Tokyo, Japan) is recommended. It allows to check the status of the protoplasts directly in the Petri dish.
10. To separate protoplasts from the majority of undigested biomass, filter the mixture from the Petri dish through the polyester (PET) mesh (pluriSelect) with a pore size of 40 μm.
11. Transfer the filtrate (containing protoplasts and short filaments) back to the Petri dish from Step 10. Seal the Petri dish with Parafilm and cover it with aluminium foil.
12. Incubate the Petri dish containing the filtrate on the rotary shaker for another 15 min (30–40 rpm shaking, room temperature). This step allows enzymatic digestion of the majority of remaining filament fragments.
13. To separate protoplast from the residual undigested filaments, filter once more through the polyester (PET) mesh (pluriSelect) with a pore size of 15 μm.
14. Since many protoplasts remain attached to the Petri dish, rinse the Petri dish with an additional 3 mL 0.5 M mannitol and filter the suspension through the same mesh as used in Step 13.
15. To separate protoplasts from the remaining cell wall debris, refilter the filtrate from the Step 13 through the polyester (PET) mesh (pluriSelect) with a pore size of 10 μm.
16. To wash away the rest of driselase, split the filtered protoplast suspension into two 15 mL glass Screw Cap Culture Tubes.
17. Centrifuge at 50 × g for 10 min with the acceleration and brake set to 3 (Eppendorf Centrifuge 5810R, rotor A-4-62).
18. Discard the supernatant.
19. Resuspend the protoplasts in 5 mL 0.5 M mannitol by gently rotating the tubes.
20. Repeat Steps 17 and 18.
21. Resuspend the protoplasts in 5 mL 0.5 M mannitol by gently rotating the tubes and combine the contents of both tubes.
22. Repeat Steps 17 and 18.
23. Resuspend the protoplasts in 4 mL RM 10 and set aside a small aliquot (about 400 μL) of the protoplast suspension for quantitative and qualitative assessment of protoplasts.
24. Check the viability of protoplasts using the light microscope (alternatively, use the fluorescein diacetate dye (FDA [40]). Determine protoplast density using a hemocytometer [41] or measure the OD_750_. Confirm the loss of cell wall using Calcofluor white M2R [42].
  Note: In total 15 protoplast isolations have been performed based on this protocol. The viability of protoplast was regularly about 95%. Protoplast density varied from 3–4 × 10^6^ protoplasts/mL. Protoplasts showed no calcofluor white fluorescence.

### Protoplast regeneration

Next, we established an efficient protocol for regeneration of the isolated protoplasts, i.e. cell wall regeneration and restoration of the original morphology. This was done in three steps.

**Fig. 5:**
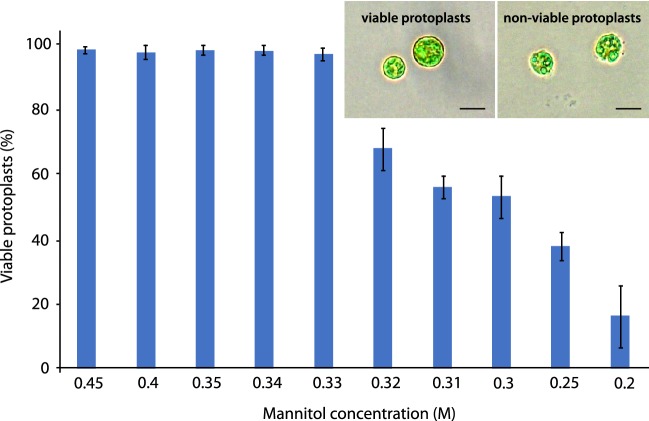
Effect of mannitol concentration on the viability of protoplasts. Bars represent ± SD from the mean of 3 replicates consisting of 1000 protoplasts per replicate. The graph is accompanied with light microphotographs showing the viable and non-viable protoplasts. Scale bar = 20 μm

First, we developed a regeneration medium (RM) that consisted of GM, mannitol and CaCl_2_. For mannitol, we used a concentration of 0.33 M, which was the lowest concentration with nearly 100% survival rate of protoplasts (Fig. 5). For CaCl_2_, 10 mM was needed for > 85% protoplast survival rate, but lower concentrations were required for cell division, filament formation and branching (Table 2). Thus, to regenerate the majority of protoplasts into branched filaments, we followed a two-step procedure. Protoplasts were incubated in RM with 10 mM CaCl_2_ (RM10 medium) for 24 h and then transferred into RM with 1 mM CaCl_2_ (RM1 medium).

**Table 2:**
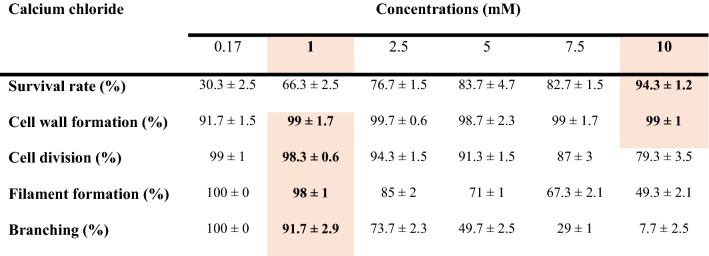
Influence of calcium chloride on protoplast regeneration in *Draparnaldia*

Concentrations used for protoplast regeneration are in bold and orange background. Values are reported as the mean ± SD for 3 replicates consisting of 500 protoplasts or filaments per replicate

**Fig. 6:**
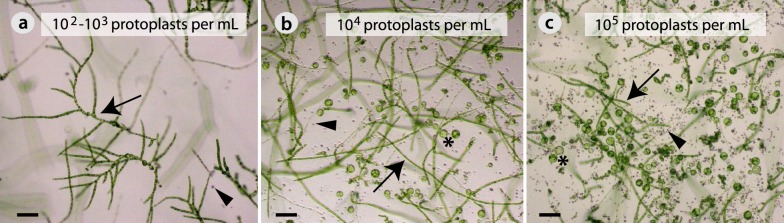
Influence of protoplast plating density on regeneration. **a** 10^2^–10^3^ protoplasts/mL. **b** 10^4^ protoplasts/mL. **c** 10^5^ protoplasts/mL. Three stages are shown: filaments (arrow), dead protoplasts (arrow head) and enlarged protoplasts (asterisks). Pictures were taken 20 days after plating. Scale bar = 80 μm

Second, we determined the optimal protoplast plating density for regeneration. All tested densities (10^2^–10^5^ protoplasts/mL) enabled regeneration into filaments (arrows in Fig. 6a–c). But, only the lowest densities (10^2^–10^3^ protoplasts/mL) allowed for branching (Fig. 6a) and were taken as the optimal plating densities for regeneration.

Third, we replaced RM by GM. Because the complete replacement of RM was too drastic (> 50% filaments died), gradual replacement (with 100% survival of filaments) was chosen for the protocol (see Steps 4 and 5).

**Fig. 7:**
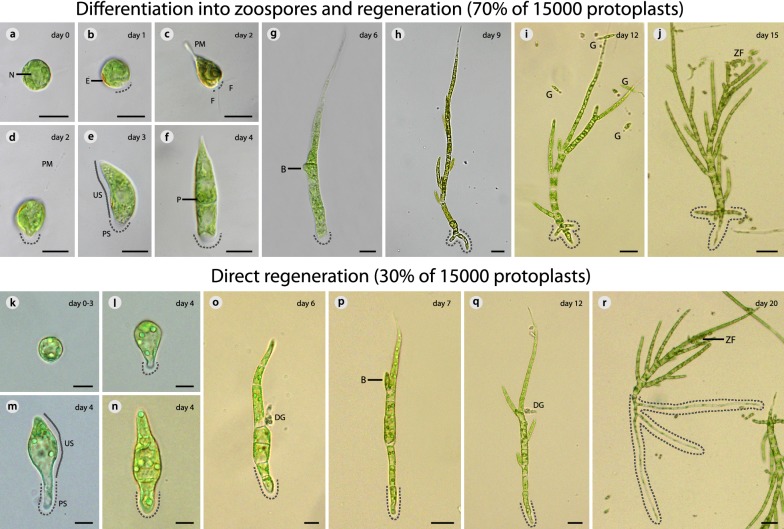
Two modes of *Draparnaldia* protoplast regeneration. **a**–**j** Differentiation into zoospores and regeneration. **a** Protoplast. **b** Protoplast with eyespot. **c** Zoospore. **d** Attached zoospore. **e**–**i** Germination and formation of the Upright System (US) with branching and the Prostrate System (PS). **j** Mature alga. **k–r** Direct regeneration. **k** Protoplast. **l**–**q** Germination and formation of the Upright System (US) and the Prostrate System (PS). **r** Mature alga. *B* branching, *DG* dead germling, *E* eyespot, *F* flagellum, *N* nucleus, *P* pyrenoid, *PM* plasma membrane, *PS and dashed line* prostrate system, *US* upright system, *ZF* zoospore formation. Scale bar: **a**–**h**, **k**–**p** = 10 μm; **q** = 20 μm; **i, j, r** = 40 μm

Two modes of protoplast regeneration were observed: protoplasts either differentiated into zoospores (= motile reproductive bodies) and regenerated (Fig. 7a–j) or they regenerated directly (Fig. 7k–r). The resulting protocol allowed for a successful regeneration of > 90% protoplasts within 15 (via zoospores) or 20 days (direct regeneration). In both cases, the regenerated filaments resembled the original *Draparnaldia* morphology and were able to reproduce (ZF in Fig. 7j, r).

#### Protocol for protoplast regeneration

1. Pre-incubate protoplasts for 24 h in 4 mL RM10 in a glass Screw Cap Culture Tube. Gently resuspend the protoplasts at least 3 ×/day.
  Note: Do not pre-incubate the protoplasts in Petri dishes or plates. Unlike protoplasts from embryophytes, those of *Draparnaldia* attach to the surface and cannot be removed without damage. Protoplasts can be kept in RM10 up to 5 days, after that the regeneration rate decreases to about 70%.
2. Adjust the density of protoplasts in RM1 medium to 10^2^–10^3^/mL.
  Note: Use cut filter tips for manipulation with protoplasts. This will eliminate their damage.
3. Incubate protoplasts in RM1 until the first branches occur. Importantly, protoplasts will not regenerate in a glass tube, they must be incubated in a Petri dish or in a culture plate.
  Note: Dish (plate) should not be filled to more than 50% to allow for proper gas exchange. Seal the dish (plate) with Parafilm to avoid contamination and evaporation.
4. As soon as branching is observed (usually 5–7 days after incubation in RM1) remove 50% of the RM and add the same volume of GM.
  Note: The regenerated filaments are firmly attached to the surface using their Prostrate System (PS and dashed line in Fig. 7). Therefore, the majority of them will not be washed out during the media exchange.
5. Repeat Step 4 on the next day.

### Transfection of protoplasts

**Fig. 8:**
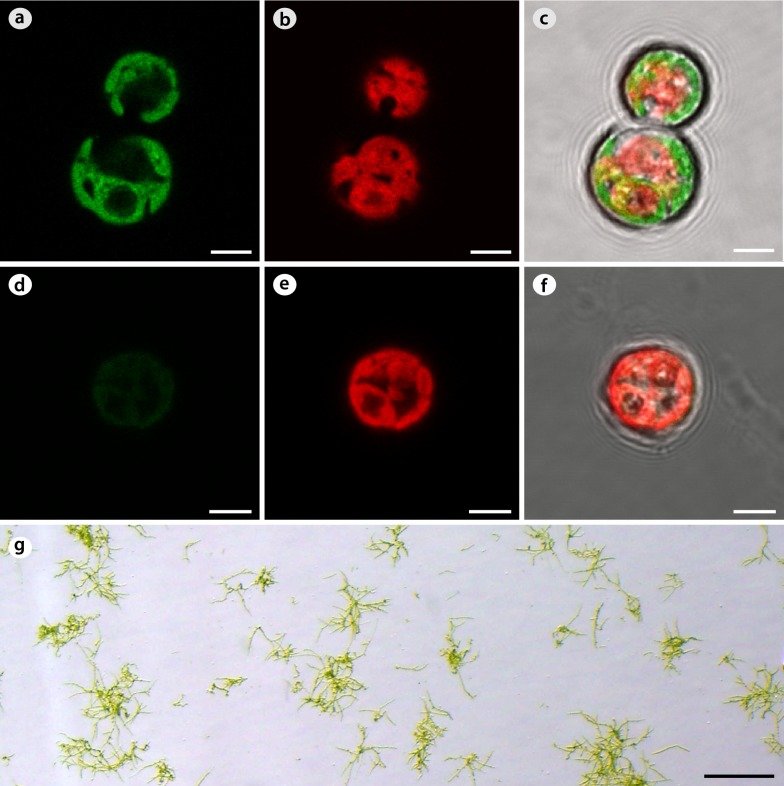
Expression and regeneration of electroporated protoplasts. **a**–**c** Expression in protoplasts. **a** YFP channel (488 nm). **b** Chlorophyll channel (561 nm). **c** Merged. Pictures were taken 3 days after electroporation. **d**–**f** Negative control. **d** YFP channel (488 nm). A weak background fluorescent from the chloroplast is visible. **e** Chlorophyll channel (561 nm). **f** Merged. Pictures were taken 3 days after electroporation. **g** Regenerated filaments from electroporated protoplasts. A picture taken 15 days after electroporation at 300 V. Scale bar: **a**–**f** = 5 μm; **g** = 500 μm

Next we examined whether protoplasts can be transfected. The transfection was performed using a Hsp70A-Rbc S2::Zeo::FMDV2A::YFP construct (pChlamy_4-eYFP, see Fig. 3) by electroporation at three different voltages. The reporter gene expression was observed in the majority of living protoplasts (> 80%) electroporated at 300 V (Fig. 8a–c), but not at 400 and 600 V. Also a negative control did not show any expression, only a weak background fluorescent from the chloroplast was visible (Fig. 8d–f). The expression was transient as it disappeared about 8 days after transfection. The survival percentage of electroporated protoplasts depended on the voltage. About 83% of protoplasts survived 300 V, 70% survived 400 V, and only 50% survived 600 V. All surviving protoplasts could be fully regenerated (Fig. 8g).

#### Protocol for transfection of protoplasts

1. Pre-incubate protoplasts for 24 h in 4 mL RM10 in a glass Screw Cap Culture Tube. Gently resuspend the protoplasts at least 3 ×/day.
2. Measure protoplast density by OD_750_: Pipette 900 μL RM10 into the 1.5 mL semi-micro cuvette (12.5 × 12.5 × 4.5 mm). Add 100 μL of the protoplast suspension and stir gently with a cut filter tip. Perform measurement and throw the sample away.
  Note: Using cut filter tips for manipulation with protoplasts will eliminate their damage.
3. Resuspend protoplasts from Step 1 in RM10 to a final OD_750_ of 0.5.
  Note: Again, use cut filter tips for manipulation with protoplasts.
4. Incubate protoplasts on ice for 20 min.
5. Pipette 400 μL of the cooled protoplast suspension with cut filter tip into the 1.5 mL pre-chilled microtube.
6. Add approximately 1 μg of linearized plasmid and stir gently with a cut filter tip.
7. Using a cut filter tip, transfer the mixture to a pre-chilled 0.4-cm electroporation cuvette with a 2 mm gap.
8. Perform electroporation (electrical pulse 300 V, 2 ms).
9. After electroporation, incubate protoplasts on ice for additional 3 min. While waiting, prepare a Petri dish for protoplast regeneration: Place three sterile square coverslips on the bottom of a 60 × 15 mm Petri dish and add 7 mL of RM1.
10. Using a cut filter tip, pipette the protoplast suspension out of the cuvette and placed it on the top of coverslips.
  Note: Note that if coverslips are not applied protoplasts attach to the surface of the Petri dish and cannot be collected for microscopy without damage.
11. Seal the Petri dish with Parafilm to prevent evaporation during regeneration.
12. Regenerate protoplasts under standard conditions as described in Ref. [13].
  Note: Incubation of protoplasts in the dark is not required.

### Selective agents

Finally, to see if the zeocin resistance in the pChlamy_4 plasmid might be suitable for selection, we evaluated the sensitivity of regenerated protoplasts (the wild-type) to zeocin. In addition, we also tested several other antibiotics and herbicides commonly used for plant and algal selection. Seven out of ten agents tested (including zeocin) were able to kill *Draparnaldia*. Herbicides were effective within 1 week and antibiotics within 2 weeks. Three antibiotics were ineffective, having no effect on growth. An overview of tested antibiotics and herbicides and the minimum inhibitory concentrations of the effective ones is provided in Table 3.

**Table 3:**
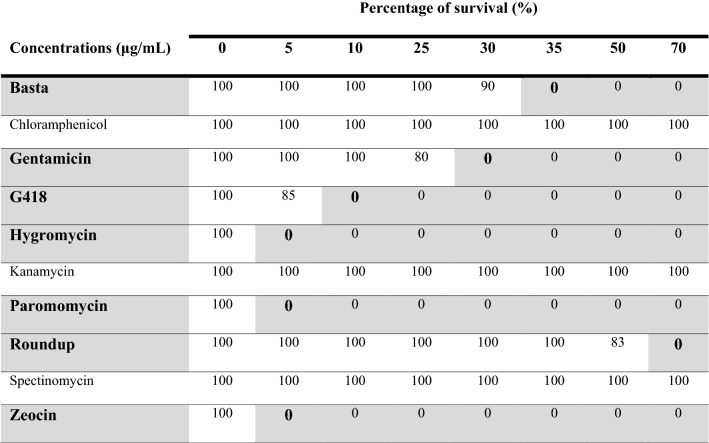
Effects of different selective agents on *Draparnaldia* (the wild-type)

The effective antibiotics and herbicides are in bold and grey background. Note that dead filaments were recognized by pale color. Percentage survival of *Draparnaldia* filaments based on 3 replicates

## Discussion

In this study we developed a protoplast transformation protocol for *Draparnaldia*.

The yield of protoplasts from a single isolation (10^6^ protoplasts) was comparable with protoplast yields reported for well-established model organisms, such as *Physcomitrella patens* [16] or *Arabidopsis thaliana* [43, 44]. Similarly to those organisms, a high protoplast yield could only be achieved when young plant/algal material was used (see “Results”). Probably older plant/algal cells have more complex rigid cell walls, which are more resistant to enzymatic digestion [45–47]. To generate young homogenous biomass, we recommend to grow *Draparnaldia* in aerated liquid culture (Additional file 2). The combination of fresh medium and aeration transfers the majority of cells (more specifically their protoplasts) into zoospores that give rise to new filaments. The intensity of aeration should be adjusted to ensure homogeneous culture suspension, which is important for equal growth of filaments. Note, that *Draparnaldia* can also theoretically be transformed by using zoospores (flagellates lacking a cell wall), like gametes of seaweed *Ulva* [19]. However, *Draparnaldia* zoospores do not have a strong phototaxis and stay motile for only a few minutes before attaching to the substrate. This makes their collection and purification very difficult and thus prevents them from being used as an efficient source for transformation.

Protoplast regeneration was based on the same parameters as regeneration in other plant/algal organisms [16, 19, 46, 48–54]. These parameters were: optimized osmoticum, calcium chloride and plating density of protoplasts (see “Methods” section). Note, that similar to mosses, no addition of plant hormones was needed. Importantly, *Draparnaldia* protoplasts can be regenerated in Petri dishes or multiwell culture plates, but not in glass tubes. This problem does not seem to be related to the glass material itself (protoplasts can regenerate on coverslips, see “Results”), but rather to some other conditions such as suboptimal light or gas exchange. Another interesting observation was that the majority of protoplasts differentiated into zoospores prior to their regeneration (see “Results”). In fact, this behaviour mimics natural development of *Draparnaldia* (compare Figs. 2 and 7) and it can be explained by using young biomass for protoplast isolation. Young biomass is known to be favorable for zoospore formation [13, 55]. Moreover, *Draparnaldia* zoospores can be easily induced by abiotic stresses, such as medium exchange [13, 56]. Therefore, it is likely that the observed zoospores were either initiated during protoplast isolation (probably when GM was replaced by mannitol) or when protoplasts were transferred into RM.

We also showed that *Draparnaldia* protoplasts are capable of being transiently transfected by electroporation. Thus, the next goal will be to determine whether they can be stably transformed. Success in achieving this goal will require optimization of the electroporation as well as testing alternative approaches of transgene delivery. This includes alternative procedures for direct uptake of DNA by protoplasts [19, 25], particle bombardment [57, 58] as well as *Agrobacterium*-mediated conjugative transformation [59]. Also, it will be important to determine whether stable transformation occurs via homologous recombination (like in *Physcomitrella*, [60, 61]) or random integration (like in most land plants, [62]).

Finally, we demonstrated that regenerated protoplasts (= the wild type) are sensitive to several commonly used selective agents (see “Results”. This sensitivity to multiple agents is advantageous for at least two reasons. (1) It might allow a number of molecular applications, including gene stacking, generation of T-DNA mutant libraries and subsequent mutant complementation, and assays where multiple reporter genes are required such as BiFC [63, 64]. (2) It also suggests that many available antibiotic/herbicide reporter genes could potentially be used for *Draparnaldia* transformation.

## Conclusions

Here we present the first protoplast transformation protocol for *Draparnaldia* sp.—the emerging chlorophyte model for comparative analysis with early streptophytes. The protocol yields 10^6^ protoplasts per isolation. It allows full regeneration of > 90% of protoplasts and enables protoplast transient transfection by electroporation. To further expand this toolset, we also identified selective agents that are suitable for selection of future *Draparnaldia* transformants. This protocol opens a way for functional analyses in *Draparnaldia*. It also provides the first step towards establishing stable transformation in *Draparnaldia*. Although our protocol has been established for *Draparnaldia*, we believe that it can be used as a reference to develop transformation protocols in other chlorophyte as well as streptophyte filamentous algae.

## Additional files

**Additional file 1.** List of chemicals (Supplement 1a) and equipment (Supplement 1b).

**Additional file 2.** Cultivation of *Draparnaldia*. This file contains information on Growth Medium (GM), establishment of actively growing culture, scaling up biomass and establishment of long-term cultures. It also includes instructions about how to assemble and inoculate flasks for aerated liquid culture. Finally, it provides advices how to keep the culture axenic.

## Data Availability

All data generated or analysed during this study are included in this published article and its additional files.
