# Supplementary material for: Regeneration and transient gene expression in protoplasts of Draparnaldia (chlorophytes), an emerging model for comparative analyses with basal streptophytes
Source: Plant Methods. 2019 Jul 12;15:74. doi: 10.1186/s13007-019-0460-6 (PMC6624896; doi:10.1186/s13007-019-0460-6)
Supplement: Supplementary file 1 — Additional file 1. List of chemicals (Supplement 1a) and equipment (Supplement 1b). [file 13007_2019_460_MOESM1_ESM.pdf]

Supplement 1a

Chemicals

- Basta – Glufosinate-ammonium (Bayer, Cat. no. 6943)
- (+)-Biotin ($C_{10}H_{16}N_2O_3S$) (SERVA, Cat. no. 15060)
- Boric acid (H_3BO_3) (ROTH, Cat. no. 6943)
- Calcium chloride dihydrate ($CaCl_2 \cdot 2\ H_2O$) (ROTH, Cat. no. 5239)
- Calcofluor white stain (Sigma-Aldrich, Cat. no. 18909-100ML-F)
- Chloraphenicol ($Cl_2CHCONHCH(CH_2OH)CH(OH)C_6H_4NO_2$) (Sigma-Aldrich, Cat. no. C0378-25G)
- Cobalt(II) nitrate hexahydrate ($Co\ (NO_3)_2 \cdot 6\ H_2O$) (Merck, Cat. no. 102554)
- Copper(II) sulfate pentahydrate ($CuSO_4 \cdot 5\ H_2O$) (Merck, Cat. no. 102790)
- Deionized, distilled water (ddH2O)
- D-mannitol ($C_6H_{14}O_6$) (ROTH, Cat. no. 4175)
- Driselase from Basidiomycetes sp. (Sigma-Aldrich, Cat. no. D8037)
- Ethanol, TechniSolv (VWR, Cat. no. 85830.360)
- GeneArt ™ Chlamydomonas Protein Expression Vector (Invitrogen/Thermo Fisher Scientific, Cat. no. A24231)
- Geneticin disulfat (G418) solution (ROTH, Cat. no. CP11.2)
- Gentamicin sulphate (Duchefa Biochemie, Cat. no. G0124.0025)
- Hydrochloric acid fuming 37 % (HCl) (ROTH, Cat. no. 4625)
- Hygromycin B (Invitrogen/Thermo Fisher Scientific, Cat. no. 10687010)
- Iron(II) Sulfate Heptahydrate ($FeSO_4 \cdot 7\ H_2O$) (Merck, Cat. no. 103965
- Kanamycin sulphate monohydrate ($C_{18}H_{36}N_4O_{11}.H_2SO_4.H_2O$) (Duchefa Biochemie, Cat. no. G0126.0025)
- Lugol's solution (diluted iodine-potassium iodide solution) (Merck, Cat. no. 109261)
- Magnesium sulfate heptahydrate ($MgSO_4 \cdot 7\ H_2O$) (Merck, Cat. no. 105886)
- Manganese(II) chloride ($MnCl_2 \cdot 4\ H_2O$) (Sigma-Aldrich, Cat. no. M3634)
- Molybdenum(VI) oxide (MoO_3) (Merck, Cat. no. 100403)
- Niacinamide ($C_6H_6N_2O$) (Sigma-Aldrich, Cat. no. N5535)
- Paromomycin sulfate salt ($C_{23}H_{45}N_5O_{14} \cdot xH_2SO_4$) (Sigma-Aldrich, Cat. no. P9297)
- Potassium dihydrogen phosphate (KH_2PO_4) (ROTH, Cat. no. 3904)
- Potassium hydroxide (KOH) (Merck, Cat. no. 105033)
- Potassium phosphate dibasic trihydrate ($K_2HPO_4 \cdot 3\ H_2O$) (Merck, Cat. no. 105099)
- Roundup (Bayer)
- ScaI-HF restriction enzyme (New England BioLabs, Cat. no. R3122S)
- Sodium chloride (NaCl) (AppliChem, Cat. no. 3597)
- Sodium hydroxide (NaOH) (Merck, Cat. no. 106498)
- Sodium nitrate ($NaNO_3$) (ROTH, Cat. no. A136)
- Spectinimycin HCl pentahydrate ($C_{14}H_{24}N_2O_7.2HCl.5H_2O$) (Duchefa Biochemie, Cat. no. S0188.0025)
- Thiamine·HCl ($C_{12}H_{17}ClN_4OS \cdot HCl$) (SERVA, Cat. no. 36020)
- Triplex II (Ethylenedinitrilotetraacetic acid) ($C_{10}H_{16}N_2O_8$) (Merck, Cat. no. 108417)
- Sulfuric acid 96% (H_2SO_4 conc.) (Merck, Cat. no. 100714)
- Zinc Sulfate Heptahydrate ($ZnSO_4 \cdot 7\ H_2O$) (Merck, Cat. no. 108883)
- Vitamin B_{12} ($C_{63}H_{88}N_{14}O_{14}PCo$) (SERVA, Cat. no. 38310)
- Zeocin (Invitrogen/Thermo Fisher Scientific, Cat. no. R25001)

Supplement 1b

Equipment

General equipment

- Aluminium foil (VWR, Cat. no. 291-0047)
- Autoclave

- Balance for weighing from 0.1 g to 500 g
- Beaker 200 and 2000 mL
- Clean bench
- Gloves
- Growth chamber
- Inverted cell culture microscope (e.g. CKX41, Olympus, Tokyo, Japan)
- Magnetic stirrer and Teflon magnetic stirring bar
- Measuring cylinders (50, 100 and 1000 mL)
- pH meter
- Stainless-steel spatula (or spoon, for weighing chemicals)

Equipment for culturing

- Aluminium cap (VWR, Cat. no. SCUT 362 1313)
- Aquarium pump
- Cotton plugs = stopper cellulose (VWR, Cat. no. 391-0381, 391-8922)
- Cotton wool (VWR, Cat. no. 115-2521)
- Erlenmeyer flasks 50, 100, 250, 500 and 1000 mL (VWR, Cat. no. 214-1130, 214-1131, 214-1132, 214-1133 and 214-1134)
- Gas burner
- Glass pipette 5 mL (VWR, Cat. no. 612-1133)
- Screw tubing clamp (VWR, Cat. no. 229-0350)
- Reagent bottle with screw cap 50, 100, 250, 500 and 1000 mL (VWR, Cat. no. 215-1591, 215-1592, 215-1593, 215-1594 and 215-1595)
- Tubing, autoclavable (VWR, Cat. no. 228-0720)
- Vent filters ACRO 50 with 0.2 µm PTFE membrane (VWR, Cat. no. 514-4109)

Equipment for protoplast isolation, regeneration and electroporation

- Autoclavable forceps
- BioSpectrometer basic (Eppendorf)
- Biosphere Filter Tips 100-1000 µL (Sarstedt, Cat. no. 70.762.211)
- Biosphere Filter Tips 2-200 µL (Sarstedt, Cat. no. 70.760.211)
- Biosphere Filter Tips 0.5-20 µL (Sarstedt, Cat. no. 70.1116.215)
- Bright-Line Hemacytometer (Sigma-Aldrich, Cat. no. Z359629)
- Centrifuge with a swinging bucket rotor (Eppendorf 5810 R)
- Cover glasses, square (VWR, Cat. no. 631-1567)
- Disposable cuvettes, 1.5 mL semi-micro, 12.5 x 12.5 x 45 mm (BRAND, Cat. no. 759115)
- Electroporator (Eppendorf, 2510)
- Electroporation cuvette 2mm, 400 µL, Eppendorf (Sigma-Aldrich, Cat. no. Z606855)
- Eppendorf pipettes 10, 200, 1000 µL (VWR, Cat. no. 76121-358)
- Falcon 50 mL (Sarstedt, Cat. no. 62.547.254)
- Falcon 15 mL (Sarstedt, Cat. no. 62.554.502)
- Filter tips 200 µL (Sarstedt, Cat. no. 70.760.211)
- Filter, Filtropur S 0.2 µm (Sarstedt, Cat. no. 83.1826.001)
- Haemocytometer and coverslips to fit
- Micro tubes 1.5 mL (Sarstedt, Cat. no. 72.690.001)
- Micro tubes 2 mL (Sarstedt, Cat. no. 72.695.500)
- Parafilm (VWR, Cat. no. 291-1212)
- Petri dishes 100 x 20 mm (Sarstedt, Cat. no. 83.3902)
- Petri dishes 60 x 15 mm (Sarstedt, Cat. no. 83.3901)
- Pipetus - battery powered pipette filler (e.g Hirschmann)
- pluriStrainers, mesh size of 40 µm, 15 µm and 10 µm (pluriSelect, Cat. no. 43-50040-51, 43-50015-03 and 43-50010-03)
- PYREX™ Screw Cap Culture Tubes (Fisher Scientific, Cat. no. 15-932H)

- Shaker (Heidolph, Rotamax 120)
- Serological pipettes 10 mL (Sarstedt, Cat. no. 86.1254.001)
- Serological pipettes 5 mL (Sarstedt, Cat. no. 86.1253.001)
- Syringe 50 mL (Sarstedt, Cat. no. 94.6077.137)
- TC-Plate 6 well, standart, F (Sarstedt, Cat. no. 83.3920)
- TC-Plate 24 well, standart, F (Sarstedt, Cat. no. 83.3922)
