# Supplementary material for: Regeneration and transient gene expression in protoplasts of Draparnaldia (chlorophytes), an emerging model for comparative analyses with basal streptophytes
Source: Plant Methods. 2019 Jul 12;15:74. doi: 10.1186/s13007-019-0460-6 (PMC6624896; doi:10.1186/s13007-019-0460-6)
Supplement: Supplementary file 2 — Additional file 2. Cultivation of Draparnaldia. This file contains information on Growth Medium (GM), establishment of actively growing culture, scaling up biomass and establishment of long-term cultures. It also includes instructions about how to assemble and inoculate flasks for aerated liquid culture. Finally, it provides advices how to keep the culture axenic. [file 13007_2019_460_MOESM2_ESM.pdf]

Cultivation of *Draparnaldia*

Growth medium (GM)

Growth medium is based on Bold's Basal Medium (BBM), stock solutions No 1 – 7 [1]. To this, four vitamins are added (stock solution No 8).

No	Components	Stock solution	Addition per litre of culture medium	Final concentration
1	$NaNO_3$<br>$K_2HPO_4 \cdot 3\ H_2O$<br>KH_2PO_4<br>NaCl	25 g/L dH_2O<br>9.8 g/L dH_2O<br>17.5 g/L dH_2O<br>2.5 g/L dH_2O	10 mL	2.94 mM<br>0.43 mM<br>1.29 mM<br>0.43 mM
2	$CaCl_2 \cdot 2\ H_2O$	2.5 g/L dH_2O	10 mL	0.17 mM
3	$MgSO_4 \cdot 7\ H_2O$	7.5 g/L dH_2O	10 mL	0.3 mM
4	Triplex II<br>KOH	50 g/L dH_2O<br>31 g/L dH_2O	1 mL	17.10 mM<br>55.30 mM
5	$FeSO_4 \cdot 7\ H_2O$<br>H_2SO_4 conc.	4.98 g/L dH_2O<br>1 mL	1 mL	0.179 mM
6	H_3BO_3	11.42 g/L dH_2O	1 mL	18.5 mM
7	Trace elements (autoclave to dissolve):<br>$ZnSO_4 \cdot 7\ H_2O$<br>MoO_3<br>$CuSO_4 \cdot 5\ H_2O$<br>$Co\ (NO_3)_2 \cdot 6\ H_2O$<br>$MnCl_2 \cdot 4\ H_2O$	8.82 g/L dH_2O<br>0.71 g/L dH_2O<br>1.57 g/L dH_2O<br>0.49 g/L dH_2O<br>1.44 g/L dH_2O	1 mL	0.307 µM<br>4.93 µM<br>6.29 µM<br>1.68 µM<br>7.28 µM
8	Vitamin solution (prepare 100x pre-stock solution, adjust pH to7, store it at - 20 for up to 10 years):<br>Vitamin B_{12}<br>(+)-Biotin<br>Thiamine·HCl<br>Niacinamide	0.2 mg/L dH_2O<br>1 mg/L dH_2O<br>100 mg/L dH_2O<br>0.1 mg/L dH_2O	3 mL	0.6 µg<br>3 µg<br>300 µg<br>0.3 µg
Adjust pH to 6.6 with NaOH/HCl and autoclave. The autoclaved medium as well as stock solutions can be stored at 4 °C, up to several months.				

Cultivation

Draparnaldia is fast growing, easy to culture and its culturing requires no previous experience with algae. Nevertheless, a few standard microbiological rules need to be followed to keep cultures axenic. These rules together with other useful tips on biomass production and culture maintenance are provided below. Culturing of *Draparnaldia* consists of 3 main parts: Establishment of actively growing culture, Scaling up biomass and Establishment of long-term cultures.

<u>Establishment of actively growing culture</u>

1. Order the axenic stock culture of *Draparnaldia* from the Central Collection of Algal Cultures (culture CCAC 6921). *Draparnaldia* will be shipped either as liquid culture (usually 10 mL) or on agar slant (macroscopically visible 'lawn').

2. While waiting for the culture, prepare the equipment for culturing, see below.
   Note: To minimize the risk of contamination, the growth medium (GM) should be directly autoclaved in the flasks and tubes, respectively.

3. Transfer 7 mL of biomass from liquid stock culture or ¾ biomass from agar stock culture into 50 mL Erlenmeyer flask containing 25 mL of GM.
   Note: Remaining biomass in the stock culture can be used for establishment of 'permanent' long-term cultures, see Steps 7-9.

4. Grow the new culture at 23 °C, 14 h light/10 h dark cycle with 10–15 µmol photons m^{-2} s^{-1} photon flux rate with Cool White and Cool Daylight fluorescent lamps. In 2 – 3 weeks, *Draparnaldia* will produce enough biomass to be scaled up.
   Note: The majority of new biomass originates from zoospores (i.e. motile reproductive bodies). Since the zoospore formation positively correlates with light/dark cycle [2, 3, 4], it is not recommended to grow Draparnaldia under permanent light.

<u>Scaling up biomass</u>

5. Transfer 25 mL of 2 – 3 weeks old culture into 250 mL Erlenmeyer flask containing 50 mL of GLM. Grow the newly established culture at the same conditions as described in Step 4.
   Note: Remaining 2 -3 week old biomass can be used for establishment of a 'working' long-term culture as described in Steps 10 – 12.

6. After 1 to 1.5 weeks, transfer the entire culture (50 or 75 mL) into 500 or 1000 mL Erlenmeyer flask containing around 200 or 500 mL of fresh GM, respectively. Grow the new culture under continuous aeration with filtered air using an aquarium pump. Other conditions remain as indicated in Step 4. For how to assemble flask for aerated culture and how to transfer the culture see below.
   Note: The aeration will accelerate algal growth and thus allow production of a large amount of young biomass within a few days. This is especially important for protoplast isolation (see main text).

<u>Establishment of long-term cultures</u>

Instead of re-purchasing of a new axenic stock culture of *Draparnaldia*, it is convenient to maintain a stock of the axenic culture directly in the laboratory. Two types of long-term cultures can be established:

'Permanent' long-term cultures
These cultures should be opened only once to prepare the next permanent stock cultures. At least 2 permanent stock cultures should be maintained at the same time to reduce the risk of losing the culture.

7. Place a small amount of biomass (visible by naked eye) in a tube with 5 mL of agar slant (GM supplemented with 1% of agar).
   Note: To reduce the possibility of contamination, the permanent long-term cultures should be established directly from the stock obtained from the Culture Collection. If the stock culture was shipped on an agar slant, a piece of biomass should be washed out of agar the using 2-3 mL of GM (with a sterile glass Pasteur pipette) and transferred into the fresh agar slant tube. The liquid GM is important for zoospore formation and thus for growth of fresh biomass for storage.

8. Grow the culture for 5 – 7 days in optimal conditions (Step 4) to obtain a sufficient amount of fresh biomass for storage.

9. After that transfer the culture to dim light (below 10 µmol photons m^{-2} s^{-1}) at low temperature (4 – 7 °C) for long-term storage.
   Note: Cultures can be kept in a healthy state for at least 1 year under these conditions.

'Working' long-term culture

These are slowly but still actively growing cultures that provide a continuous source of ready inoculum for scaling up biomass. Since the establishment of actively growing cultures is relatively time-consuming (2 – 3 weeks), it is recommended to prepare several working long-term cultures at once. Each of them can be directly scaled up whenever needed.

10. Transfer 7 mL of actively growing culture (Step 5) into a new 50 mL Erlenmeyer flask containing 25 mL of GM.

11. Grow the culture at 16 – 23 °C, 14 h light/10 h dark cycle with 10 – 15 µmol photons m^{-2} s^{-1}, with no aeration.

12. Transfer the culture every 4 (at 23 °C) or 7 weeks (at 16 °C) into fresh GM (1/4 'old' culture and 3/4 fresh medium).

References

1. https://www.ccap.ac.uk/media/documents/BB.pdf
2. Agrawal SC. Zoospore formation in the green alga *Stigeoclonium pascheri*. J Algal Biomass Utln. 2012;3(4):18–19.
3. Přibyl P. Light is a crucial signal for zoosporogenesis and gametogenesis in some green microalgae. Eur J Phycol. 2013;48(1):106–15.
4. Carl C, de Nys R, Lawton RJ, Paul NA. Methods for the induction of reproduction in a tropical species of filamentous *Ulva*. PLoS One. 2014;9(5):e97396.

Three basic rules to keep the culture axenic

Transfer of biomass must be done in a laminar flow cabinet. All material used for manipulation with biomass must be sterile. The bench as well as hands should be cleaned with 70% ethanol prior to all operations. Wear gloves.

To minimize a risk of contamination, a new growth medium should be directly autoclaved in the flasks or tubes, into which *Draparnaldia* will be cultured. This is especially important for maintaining the axenic stock cultures and for scaling up the biomass. Moreover, the mouth and neck of flasks (also pipette and a part of the tubing in the aerated liquid culture) should be wrapped by aluminium foil. In case of 50 mL flaks use the aluminium caps instead of aluminium foil.

There is a risk of contamination whenever a sterile flask or tube is opened. Therefore, the mouth and neck of the flask or tube should be passed through a flame whenever anything is added to or removed from a culture. Transfer of biomass should be done either by pouring (a large quantity) or by using a glass Pasteur or plastic serological pipette (a smaller quantity). Note that Pasteur pipettes need to be plugged with nonabsorbent cotton wool at the wide end before autoclaving to prevent potential contamination. This is important, because rubber bulbs, which are attached to Pasteur pipettes may contain contaminants. The cotton plug prevents contaminants from passing through the pipette to the culture.

How to assemble flasks for aerated liquid culture

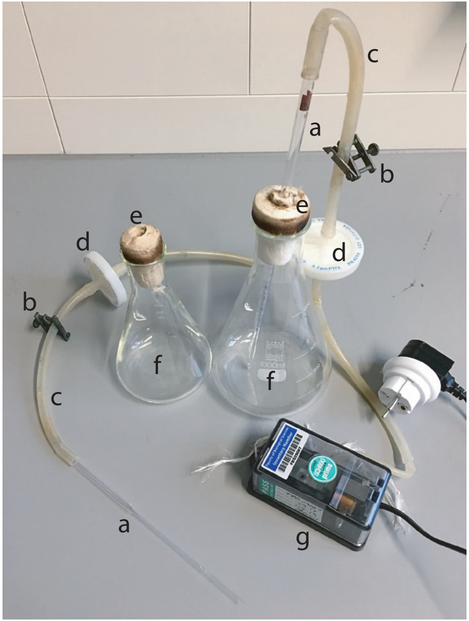

a - Pasteur pipette (for 500 mL flask),
15 mL pipette for 1000 mL flask

b - Screw tubing clamp for regulation
the aeration during culturing
(during the autoclaving it should be kept slightly opened)

c - Tubing (autoclavable)

d - Vent filters ACRO 50 with 0.2 μm
PTFE membrane

e - Cotton plug with a hole

f - 500 and 1000 mL Erlenmeyer flasks

g - aquarium pump (any type)

How to inoculate flask for aerated liquid culture

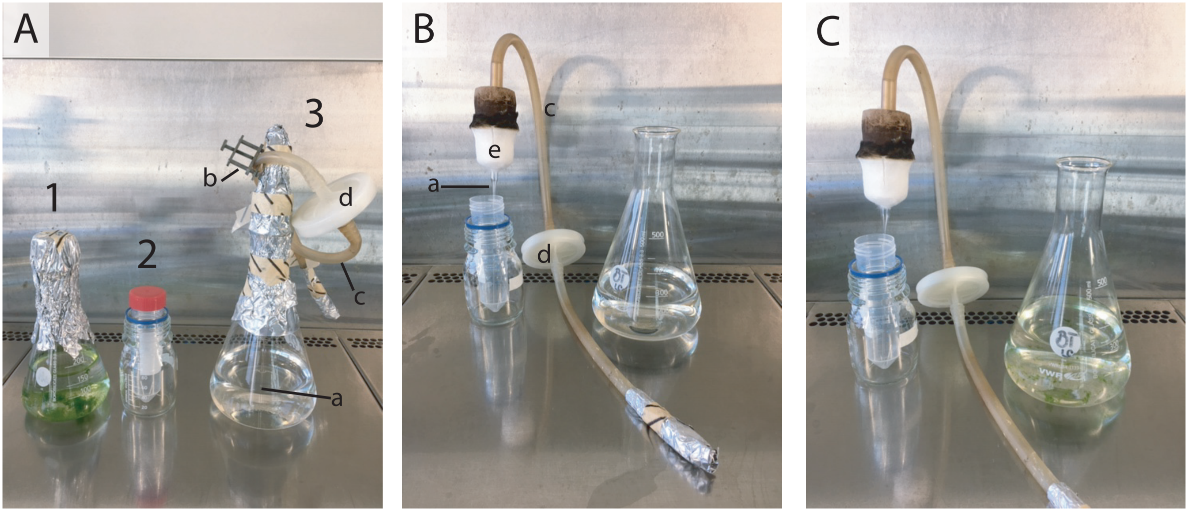

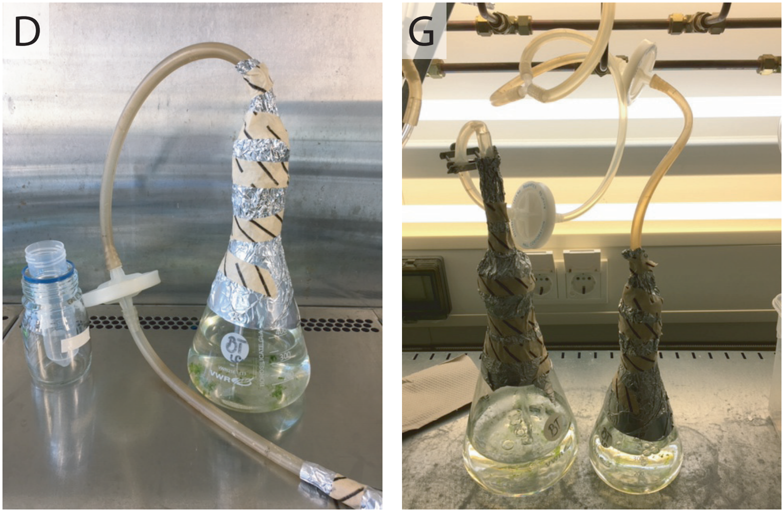

<u>1 - Flask with inoculum</u>

<u>2 - 50 mL falcon (in 50 mL schott bottle)</u>
for holding a cotton plug with
Pasteur pipette

<u>3 - 500 mL flask for culturing</u>

a - Pasteur pipette

b - Screw tubing clamp for regulation the aeration during culturing
(during the autoclaving it should be kept slightly opened)

c - Tubing (autoclavable)

d - Vent filters ACRO 50 with 0.2 μm PTFE membrane

e - Cotton plug

If Draparnaldia is grown in 1000 mL flask with 15 mL pipette, 500 mL autoclaved flask should be used for holding the pipette.
